# Selective Dielectric Metasurfaces Based on Directional Conditions of Silicon Nanopillars

**DOI:** 10.3390/nano7070177

**Published:** 2017-07-07

**Authors:** José Francisco Algorri, Braulio García-Cámara, Alexander Cuadrado, José Manuel Sánchez-Pena, Ricardo Vergaz

**Affiliations:** 1 GDAF-UC3M, Displays and Photonics Applications Group, Electronic Technology Department, Carlos III University of Madrid, Leganés, 28911 Madrid, Spain; brgarcia@ing.uc3m.es (B.G.-C.); jmpena@ing.uc3m.es (J.M.S.-P.); rvergaz@ing.uc3m.es (R.V.); 2 Laser Processing Group, Instituto de Óptica, CSIC, C/Serrano 121, 28006 Madrid, Spain; a.cuadradoconde@gmail.com

**Keywords:** nanophotonics, nanoparticles, metamaterials, silicon photonics

## Abstract

Dielectric metasurfaces based on high refractive index materials have been proposed recently. This type of structure has several advantages over their metallic counterparts. In this work, we demonstrate that dielectric metasurfaces can be theoretically designed satisfying Kerker’s zero-forward condition. This is the first time that a dielectric metasurface based on this principle has been designed. A selective dielectric metasurface of silicon nanopillars is designed to work at 632.8 nm. This structure could work both as a dielectric mirror and a reject band filter. Furthermore, by scaling up the structure, it could be possible to manufacture a terahertz (THz) dielectric mirror.

## 1. Introduction

During the past years, metamaterials have gained increasing interest [1]. Metamaterials are artificial media composed of periodic structures smaller than the wavelength of the external stimuli. In classic materials, the electromagnetic field interacts with the atoms and molecules, and depending on their structural characteristics, the material shows specific electromagnetic properties. Metamaterials enable the design of surfaces or bulks with custom electromagnetic characteristics, even producing properties that cannot be found in nature (e.g., negative refractive index), by tailoring their basic constituents. Engineered wavelength resonance, bandwidth, or scattering phase and amplitude can also be obtained [2].

The design of metamaterials and their optical characteristics can be performed by using homogenization techniques [3], by averaging the electromagnetic fields over a subwavelength volume. Some research lines about these engineered media are in a mature stage, e.g., negative-index or chiral metamaterials, however, there are still some emerging directions of research, such as quantum [4], non-linear [5], sensor [6], or active [7] metamaterials, photonic hypercrystals [8] and metasurfaces [9]. In particular, metasurfaces are the two dimensional (2D) version of 3D metamaterials. These structures produce those effects observed in three dimensional (3D) metamaterials with the unique capability to tune the light with planar components, thus obtaining a “planar photonics” device. These types of structures were studied in the past by the microwave community [10]. Recent improvements in nanofabrication processes allows transfering this knowledge to the visible spectrum. Several devices, such as super/hyper-lensing [11], cloaking [12], dielectric mirrors [13], etc., have been proposed. Specifically, the study of dielectric nanostructures has been a hot research topic in the past years [14,15]. The low absorption in the visible range and the compatibility with traditional CMOS (Complementary Metal Oxide Semiconductor) techniques of fabrication make them a suitable candidate to create flat optical components. Flat optical components have the evident advantage of compactness, which is very important for new portable and wearable photonic devices. Apart from the CMOS compatibility, the use of silicon (Si) allows considerable reductions of thickness (<100 nm), and a high aspect ratio is possible [16]. Other examples of high efficiency achieved through high aspect ratio structure can be found in vortex beam generators [17]. 

In this work, a novel approach to the design of dielectric metasurfaces is proposed. Kerker’s conditions were theoretically proposed for spherical particles in 1983 [18]. Two possible conditions were predicted, the first condition (zero backscattering) and a second one (minimum forward scattering). The latter condition appears when a destructive interference in the forward direction produces a near-cancelation of light scattering in this direction. This is not a complete cancelation as stated by Kerker, due to the fulfilment of the Optical Theorem [19]. In order to design dielectric mirrors, this condition should be satisfied. Kerker’s theory was experimentally demonstrated in the microwave spectrum in 2012 [20] and in the visible spectrum in 2013 [21]. Shortly after, a deeper minimum forward scattering for disks [22] and spheroidal [23] (tuning the shape to overlap the spectral position of the electric and magnetic dipole resonances) was found. Additionally, a recent study on silicon nanopillars (Si NPs) demonstrates an optimized forward scattering condition for an optimum aspect ratio [24]. These results demonstrate even more intense light scattering for nanopillars than those produced by previous structures (spheroids and disks). Moreover, a study of the substrate effect revealed that the use of low refractive index substrates is required in order to exploit the minimum forward condition. It was also suggested that the arrangement in metasurfaces may overcome the overall substrate effect. In this work, this hypothesis is demonstrated by looking for the minimum forward condition of Si nanopillars and arranging them in an optimized metasurface configuration. By optimizing the size of the component particles (and their distance between each other), a dielectric metasurface with a selected reflection frequency (and a maximum reflection) can be designed. This structure could be used both as a dielectric mirror and a reject band filter.

## 2. Structure and Operation Principle

**Figure 1:**
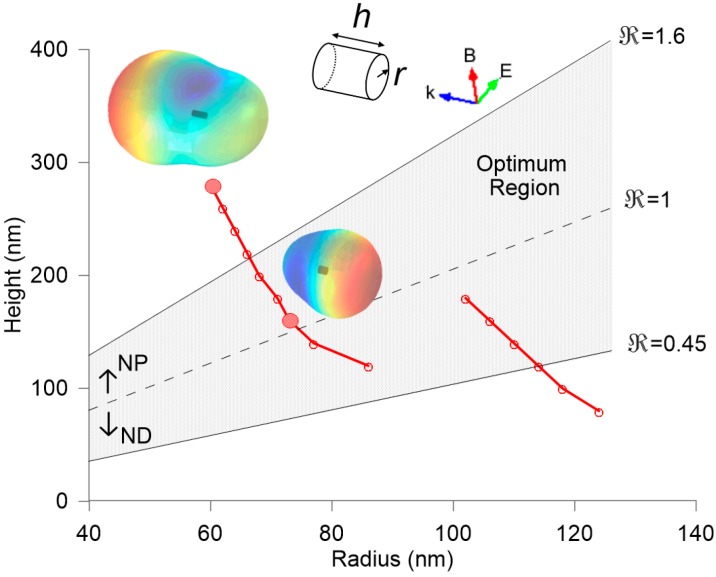
Aspect ratio (height vs. radius) of cylindrical silicon particles producing a minimum forward scattering at 632.8 nm in vacuum. The optimum observation region is delimited and shadowed. Two spatial distributions of the scattered electric field in the far-field region are included as insets, being as highest the intensity as reddish the color in a rainbow scale. Labels NP and ND refer to nanopillars and nanodisks, respectively and ℜ = *h*/2*r* is the aspect ratio.

The control of light reflection and transmission is one of the most relevant features in the design of novel metasurfaces. We already showed that an optimum aspect ratio of a silicon nanopillar can be obtained to accomplish the minimum forward scattering condition. Figure 1 reminds some of our previous results in [24] in order to clarify the optical response of the individual components of the proposed metasurface. In particular, Figure 1 plots the aspect ratios of silicon nanoparticles, either pillars or disks, where their electric and magnetic dipolar contributions satisfy the minimum forward scattering condition (red lines). The plot considers both a normal incidence on its flat surface and a certain incident wavelength (632.8 nm corresponding with commercial He-Ne lasers). A shadowed region highlights the optimum geometries producing the most directional scattering with a strongly-attenuated scattering in the forward direction. The differences of the scattered field in the far-field region between the optimum and non-optimum aspect-ratio particles can be seen in the insets. A minimum forward scattering can be observed for *h* = 160 nm and *r* = 74 nm into the optimum region (lower inset). Values out of this region, e.g., *h* = 280 nm and *r* = 60 nm (upper inset) do not present a relevant directional scattering although the minimum forward condition is still satisfied. 

**Figure 2:**
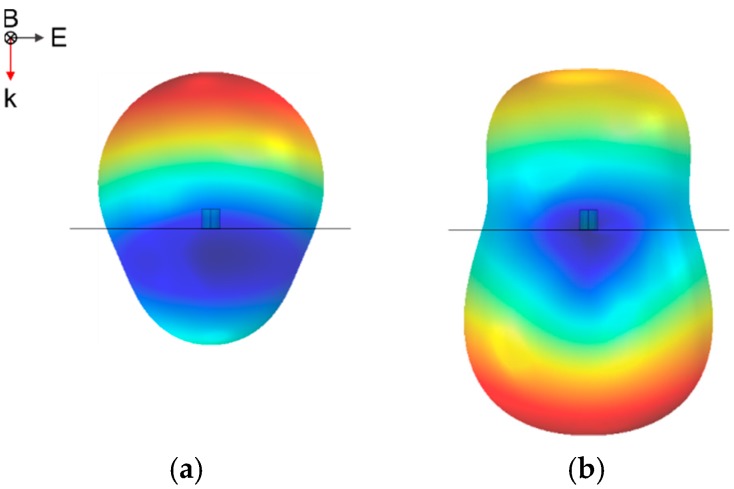
Far-field scattering (being as highest the intensity as reddish the color in a rainbow scale) of an individual nanoparticle (*h* = 160 nm, *r* = 72 nm) satisfying the minimum forward condition at *λ* = 632.8 nm, deposited over (**a**) a low refractive index substrate (aerogel) or (**b**) glass substrate.

Figure 2 shows the scattering of one of the previous nanopillars accomplishing the minimum-forward scattering condition. While Figure 2a considers a low refractive index substrate (aerogel, *n* = 1.03), Figure 2b is calculated over a glass substrate (*n* = 1.5).

**Figure 3:**
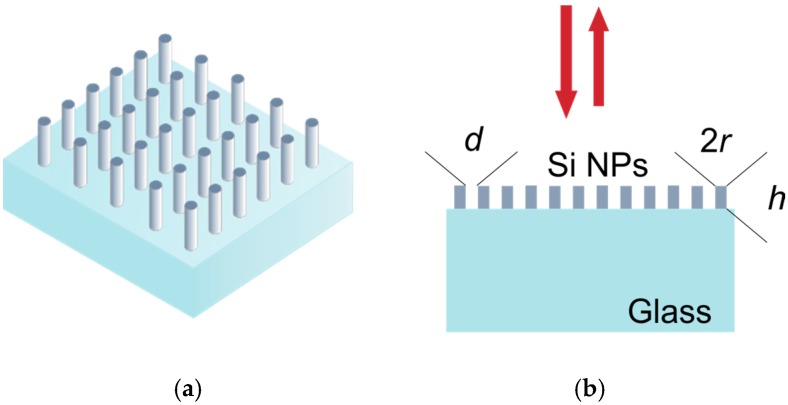
(**a**) Schematic of the silicon (Si) nanopillars metasurface, which consist of subwavelength Si nanopillars on a glass substrate; and (**b**) the geometry for the incident light vector and structural parameters of the metasurface.

As can be seen, the reduction of the refractive index contrast between the nanoparticle and the surrounding medium blurs the shape of the electric and magnetic Mie resonances excited in the particle, leading to a worse observation of the directional scattering. In order to overcome this limitation, in this work we design an ordered array (Figure 3a) of these previous particles with an optimum aspect ratio as a metasurface with reflection and transmission control of a normal incident light. The excitation of Mie resonances in individual particles are also presented in the frequency response of the whole structure. However, the intensity of the peak, particularly in the analysis of the transmission and reflection coefficients, strongly depends on the interparticle distance. Consequently, the design parameters of our device are: the radius (*r*), the height (*h*), and the distance (*d*) between NPs (Figure 3b).

Our framework is a set of numerical simulations based on Maxwell equations solved by finite element method (COMSOL^®^) (Version 5.1, COMSOL Inc., Burlington, MA, USA). The refractive indices of the Si and glass are extracted from references [25,26], respectively. A plane TE-polarized electromagnetic wave is normally incident on a silicon nanopillar array placed over a glass substrate. The symmetry of the structure at the normal incidence produces the same response for both polarizations. Estimations of the reflection coefficient and transmission coefficient are based on S-parameters (scattering parameter). The scattered-field formulation applies the weak formulation (variational calculation). COMSOL^®^ performs an eigenmode analysis on ports 1 and 2 (input and output of the light) estimating the respective electric field patterns *E*_1_, *E*_2_ of the fundamental modes on these ports. The computed electric field (*E_c_*) of the input port is the sum of the excitation and the reflected field. The reflected and transmitted powers (Equations (1) and (3), *R* and *T*, respectively) are defined through S-parameters given by Equations (2) and (4) [27]:

(1) $$R={\left|S_{11}\right|}^{2}$$

where

(2) $$S_{11}=\frac{\int_{port\;1}((E_{c}-E_{1})\cdot E_{1}^{*})dA_{1}}{\int_{port\;1}(E_{1}\cdot E_{1}^{*})dA_{1}}$$

(3) $$T={\left|S_{21}\right|}^{2}$$

where

(4) $$S_{21}=\frac{\int_{port\;2}(E_{c}\cdot E_{2}^{*})dA_{2}}{\int_{port\;2}(E_{2}\cdot E_{2}^{*})dA_{2}}$$

where *A* is the area of each port.

## 3. Results

As was mentioned, in order to observe clearly directional effects, and in particular the desired minimum forward scattering, low refractive index substrates under the nanoparticle are required [24] (see Figure 2a). This is a consequence of the excitation of the electric and magnetic Mie resonances in the particles which requires a high refractive index contrast between the particles and the surrounding medium. Nevertheless, the growing or deposition of such nanoparticles over such substrates is not an easy experimental task. Commonly, silicon or glass substrates are used in the design and fabrication of such devices, appreciably reducing the observance of resonant and directional effects. In this work a glass substrate is used because although this could dispel the minimum forward scattering condition found (Figure 2b), it will ease future fabrication. The main result of this work is the following: when nanoparticles are arranged in an array configuration forming a metasurface, the negative effect of increasing the index of the substrate may be overcome, as it will be demonstrated. This effect can be used in order to ease the creation of structures with a selected frequency of reflection.

**Figure 4:**
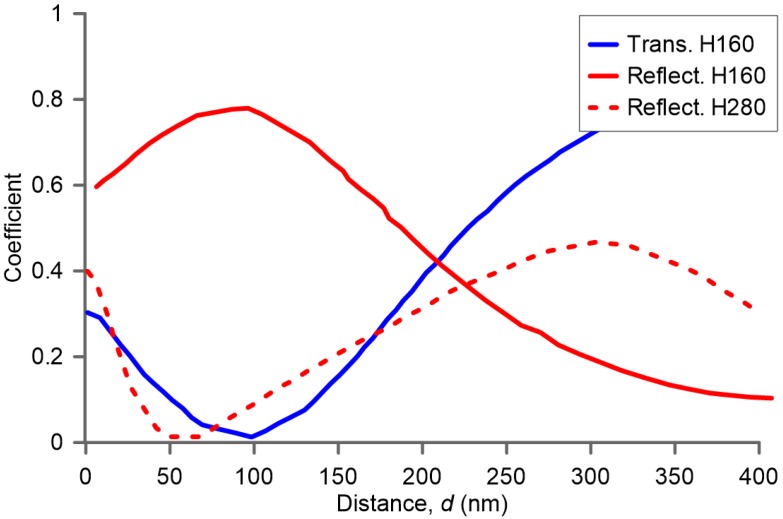
Reflected (red) and transmitted power (blue) for a metasurface of Si nanoparticles with *h* = 160 nm, *r* = 74 nm (solid line) and *h* = 280 nm, *r* = 60 nm (dashed line), as a function of distance between nanoparticles (NPs). Only transmission with *h* = 160 nm is shown.

Once the structural dimensions of each individual nanoparticle is established by using the results of Figure 1 to obtain the best directional scattering of each component, the optimum distance *d* between them in the metasurface must be determined. For this study, a numerical process determines the maximum power reflectance for a specific distance *d*. In Figure 4, it is shown how this distance affects both power reflectance and transmittance coefficients of the structure, as expected. The power coefficients (reflectance: red solid line; transmittance: blue solid line) of a metasurface composed of silicon nanopillars with an optimum aspect ratio, a height of 160 nm and a radius of 74 nm, is plotted as a function of the array constant (*d*). Regarding the optimization of minimum-forward scattering of the metasurface, a minimum transmittance is expected. A maximum reflectance, and minimum transmittance, is found for an inter-particle distance of *d* = 100 nm. The longer the distance, the lower the reflection; this is mainly due to the direct transmission of the incoming light through the nanoparticle gap. In contrast, if the minimum forward condition is not in the optimum region (Figure 4, dashed line, nanoparticles of *h* = 280 nm), the power reflectance coefficient is appreciably lower. Once the distance *d* that produces a maximum reflectance is selected, the three parameters of the configurable metasurface (*r*, *h*, *d*) are established.

**Figure 5:**
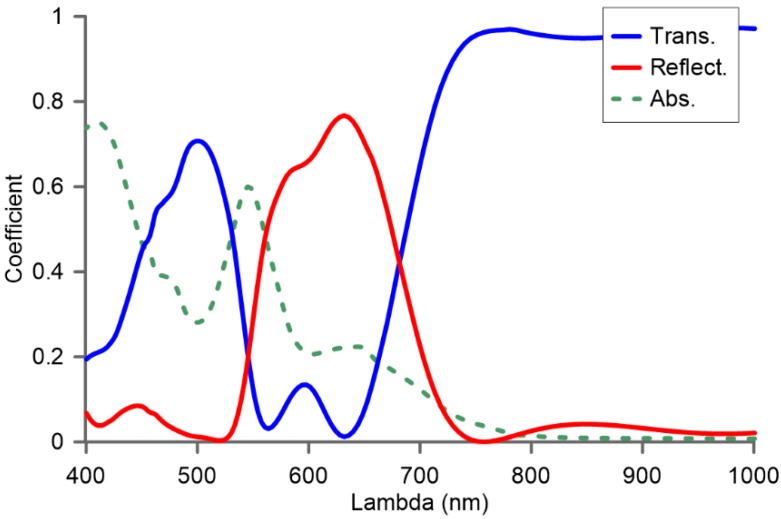
Power absorption, reflectance and transmittance coefficients for a metasurface made of Si nanopillars with *h* = 160 nm, *r* = 74 nm and distance between them of *d* = 100 nm.

Despite the complexity of the structure and the high number of different effects that govern the optical response of such a metasurface, we have spectrally characterized the selected metasurface with *d* = 100 nm. This is computed using Equations (1)–(4) and the method described in Section 2, and the results are shown in Figure 5. The power reflectance coefficient has a maximum at a wavelength *λ* = 632.8 nm, as it was designed from Figure 1 for one nanoparticle, so that the spectral performance of the whole surface maintains the spectral signature of each one of its individual components, as expected. This result demonstrates how the resonance reflection band of the metasurface can be engineered and spectrally tuned just by designing their individual components so that they satisfy the second order Kerker’s condition (minimum forward scattering). On the other hand, the high value of the power reflectance coefficient obtained at the optimized wavelength for which the metasurface was designed demonstrates the power of the optimization parameter *d*, regarding the interparticle distance, to obtain an optimized flat metasurface in terms of reflection properties. 

Finally, a remarkable result is concluded: the use of a glass substrate has not been a drawback in the reflection properties of the metasurface, as both the wavelength at which the maximum was designed to occur and the value of the power reflectance coefficient peak has been maintained in the step from nanoparticle to metasurface. Several angles have been studied (from 0° to 85°), and the result is that the wavelength at the maximum reflection for which the metasurface was optimized is maintained in the whole angular range, although the value of the power could slightly decrease.

## 4. Conclusions 

In this work, a novel approach to the design of dielectric metasurfaces is proposed and theoretically demonstrated. When optimized Si nanoparticles producing a minimum forward condition are arranged in an array configuration, the optical response of the overall structure is spectrally similar to that of each individual particle. 

The use of non-low but easy-to-obtain refractive index substrates has been shown to degrade the directional scattering for individual particles. However, in this work we have demonstrated that an array configuration with an optimum design overcomes this limitation, producing an enhanced reflection. The power reflectance coefficient magnitude is related to the inter-particle distance, and neither the peak nor the intensity have been degraded by the use of a simple glass substrate. Consequently, the distance parameter between nanoparticles can be optimized to provoke a maximum power reflectance coefficient at the designed wavelength for one individual nanoparticle. 

The experimental community should consider that this result could be scaled up to other frequency ranges, e.g., THz, in order to create extremely flat band pass filters or selective dielectric mirrors that could be easy to build.
